# Charge Berezinskii-Kosterlitz-Thouless transition in superconducting NbTiN films

**DOI:** 10.1038/s41598-018-22451-1

**Published:** 2018-03-06

**Authors:** Alexey Yu. Mironov, Daniel M. Silevitch, Thomas Proslier, Svetlana V. Postolova, Maria V. Burdastyh, Anton K. Gutakovskii, Thomas F. Rosenbaum, Valerii V. Vinokur, Tatyana I. Baturina

**Affiliations:** 1 A. V. Rzhanov Institute of Semiconductor Physics SB RAS, 13 Lavrentjev Avenue, Novosibirsk, 630090 Russia; 2 Novosibirsk State University, Pirogova str. 2, Novosibirsk, 630090 Russia; 3 The James Franck Institute and Department of Physics, The University of Chicago, Chicago, IL 60637 USA; 4 Division of Physics, Mathematics, and Astronomy, California Institute of Technology, Pasadena, CA 91125 USA; 5 Institut de recherches sur les lois fundamentales de l’univers, Commissariat de l’énergie atomique et aux énergies renouvelables-Saclay, Gif-sur-Yvette, France; 6 Materials Science Division, Argonne National Laboratory, 9700 S. Cass Ave, Argonne, IL 60439 USA; 7 Computation Institute, University of Chicago, 5735 S. Ellis Avenue, Chicago, IL 60637 USA; 8 Departamento de Física de la Materia Condensada, Instituto de Ciencia de Materiales Nicolás Cabrera and Condensed Matter Physics Center (IFIMAC), Universidad Autónoma de Madrid, 28049 Madrid, Spain

## Abstract

Three decades after the prediction of charge-vortex duality in the critical vicinity of the two-dimensional superconductor-insulator transition (SIT), one of the fundamental implications of this duality—the charge Berezinskii-Kosterlitz-Thouless (BKT) transition that should occur on the insulating side of the SIT—has remained unobserved. The dual picture of the process points to the existence of a superinsulating state endowed with zero conductance at finite temperature. Here, we report the observation of the charge BKT transition on the insulating side of the SIT in 10 nm thick NbTiN films, identified by the BKT critical behavior of the temperature and magnetic field dependent resistance, and map out the magnetic-field dependence of the critical temperature of the charge BKT transition. Finally, we ascertain the effects of the finite electrostatic screening length and its divergence at the magnetic field-tuned approach to the superconductor-insulator transition.

## Introduction

In the early 1970s, Vadim Berezinskii^1^, Michael Kosterlitz, and David Thouless^2,3^ introduced the idea of a topological phase transition in which pairs of bound vortex excitations unbind at a critical temperature *T*_BKT_. The nature of the Berezinskii-Kosterlitz-Thouless (BKT) transition is different from the standard phase transitions described by the Landau paradigm. There is no symmetry breaking associated with the onset of the order parameter, but instead there is a change in the behavior of the two-point correlation function. At *T* < *T*_BKT_ it decays algebraically, changing to exponential for *T* > *T*_BKT_. Thin superconducting films soon became the principal experimental realization for studying the BKT transition^4,5^. However, detailed investigations of the physics of the superconductor-insulator transition (SIT) in Josephson junction arrays (JJA) led to the prediction that the charge-anticharge unbinding BKT transition should appear at finite temperature on the insulating side of the SIT^6–8^. Using the framework of gauge theory, Diamantini *et al*.^9^ demonstrated that in the planar JJA, the vortex-charge duality leads to a zero-temperature quantum phase transition between a superconductor and its mirror image, a state with zero conductance, which they termed a superinsulator. The physical origin of a superinsulating state is the charge confinement due to the logarithmic interaction between the charges in two-dimensional (2D) systems^6,10,11^. In disordered superconducting films, the charge confinement on the insulating side of the SIT results from the divergence of the dielectric constant *ε* in the critical vicinity of the transition. The logarithmic interaction holds over distances $d < r\mathop{ < }\limits_{ \tilde {}}{\rm{\Lambda }}\simeq \varepsilon d$, where *d* is the thickness of the film and Λ is the electrostatic screening length^11^. This parallels the logarithmic interaction between vortices on the superconducting side, which causes the vortex binding-unbinding topological BKT transition into the superconducting state at finite temperature *T* = *T*_VBKT_^1–3^. Accordingly, logarithmic interactions between charges on the insulating side of the SIT is expected to give rise to a classical charge BKT transition into the superinsulating state with the conductance going to zero at a finite temperature *T* = *T*_CBKT_^10–12^. Applied magnetic fields can tune the SIT with high resolution, offering a window into unexplored electronic functionalities. In the critical vicinity of the SIT on the superconducting side the system should possess superinductance^13^, and at the insulating side the system is expected to be a supercapacitor due to a diverging dielectric constant^11^. This calls for a thorough study of the highly resistive state that terminates two-dimensional superconductivity at the quantum critical point whose nature remains a subject of intense research^10,14–19^.

Existing experimental data on JJA^20–22^, superconducting wire networks^23^, InO_*x*_^19^, andTiN films^18,24^ support the picture of the dual vortex-charge BKT transitions and corresponding formation of the mirror superconducting-superinsulating states. While there have been numerous experimental hallmarks of superinsulating behavior, the evidence for the charge BKT transition, with its characteristic criticality (see Eq. (1) below), has remained elusive. To address this challenge, we examine a NbTiN film, which is expected to combine the high stability of TiN films with the enhanced superconducting transition temperature *T*_*c*_ of NbN films, due to a larger Cooper pairing coupling constant as compared to TiN. We thus expect that *T*_CBKT_ is likewise enhanced, opening a wider window for observing critical behavior.

## Sample preparation

**Figure 1:**
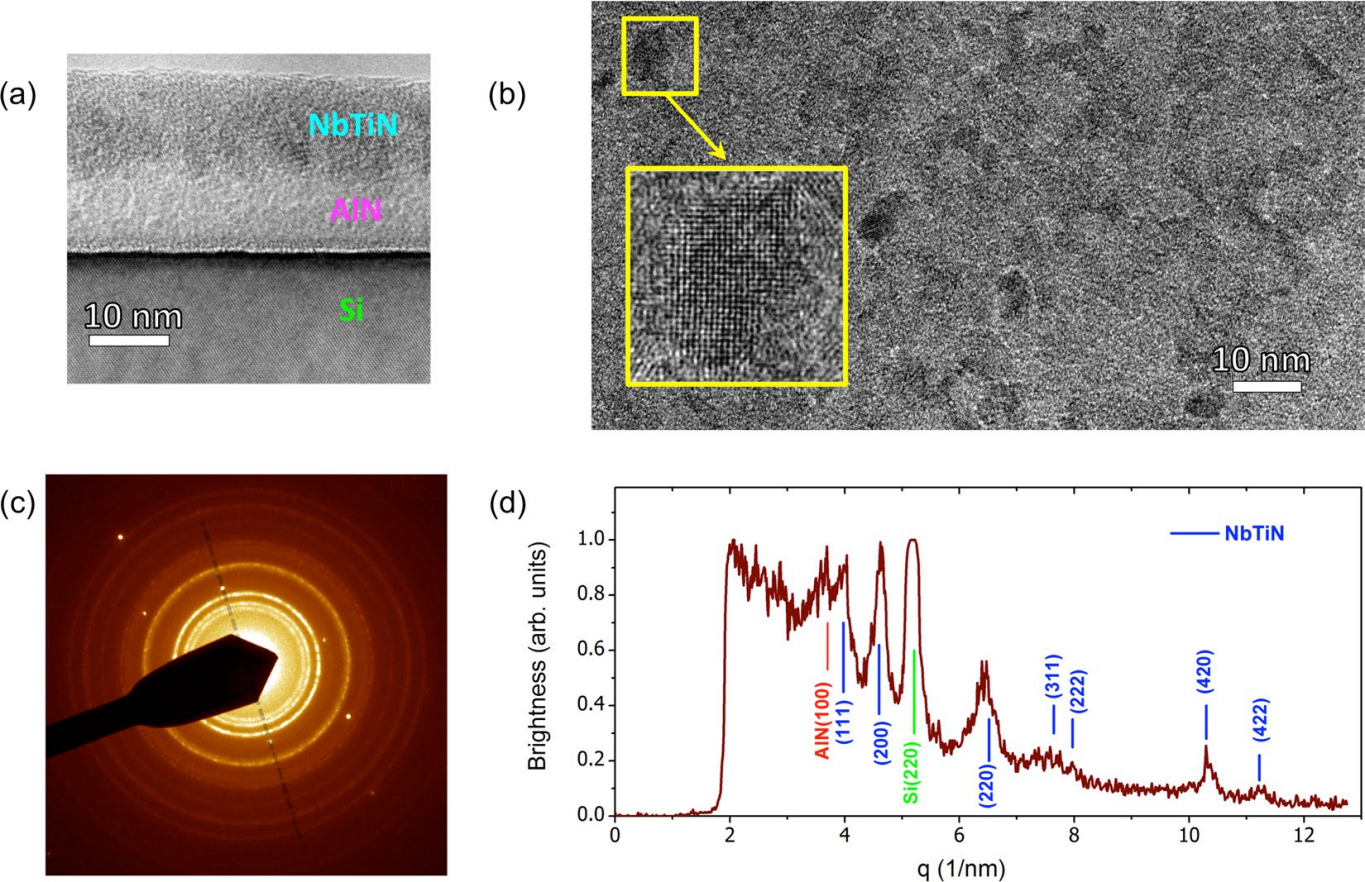
Structure and composition of a 10 nm thick NbTiN film. (**a**) Cross-section of the film from High Resolution Transmission Electron Microscopy (HRTEM). (**b**) HRTEM plan view bright field image. The yellow square shows a magnified image of one of the crystallites. (**b**) Electron-diffraction data of the film. The rings are characteristic of polycrystalline structures; the bright spots arise from the crystalline lattice of the underlying Si substrate. (**d**) Electron diffraction data from the panel (c) taken along the self-transparent dashed line in panel (c) and plotted as the intensity vs. the wavenumber. Several Bragg peaks from the NbTiN film are observed, along with the (220) peak from the Si substrate and the (100) peak from the AlN buffer layer.

To grow suitable NbTiN films, we employed the atomic layer deposition (ALD) technique based on sequential surface reaction step-by-step film growth. The fabrication technique is described in detail in the Supplemental Material. This highly controllable process provides superior thickness and stoichiometric uniformity and an atomically smooth surface^25,26^ as compared to chemical vapor deposition, the standard technique used to grow NbTiN films^27^. We used NbCl_5_, TiCl_4_, and NH_3_ as gaseous reactants; the stoichiometry was tuned by varying the ratio of TiCl_4_/NbCl_5_ cycles during growth^28^. The superconducting properties of these ultrathin NbTiN films were optimized by utilizing AlN buffer layers grown on top of the Si substrate^29^. NbTiN films of thicknesses *d* = 10, 15, and 20 nm were grown, varying only the number of ALD cycles (240, 420, and 768 cycles, respectively), with all other parameters of the ALD process held constant. We show in Fig. 1(a) a high-resolution transmission electron microscopy (HRTEM) image of the cross-section of the 10 nm thick NbTiN film. It reveals that both the AlN buffer layer and the NbTiN have a fine-dispersed polycrystalline structure. Presented in Fig. 1(b) is a plan view of a large area containing many crystallites. The densely packed crystallites have different orientations and are separated by atomically thin inter-crystallite boundaries. A statistical analysis of the image finds the average crystallite size to be approximately 5 nm. The electron diffraction data for the film are shown in Fig. 1(c). The clearly seen rings confirm a polycrystalline structure. The analysis of the diffraction data along the direction [220] of the Si substrate displayed in Fig. 1(d) reveals that the NbTiN crystallites have the same rock-salt crystal structure as both NbN and TiN. Using Vegard’s law, we find that our NbTiN film is an approximately 7:3 solid solution of NbN and TiN (See Supplemental Material for further information on the fabrication and measurement techniques, films structural, superconducting and transport parameters, and *I*–*V* characteristics).

**Figure 2:**
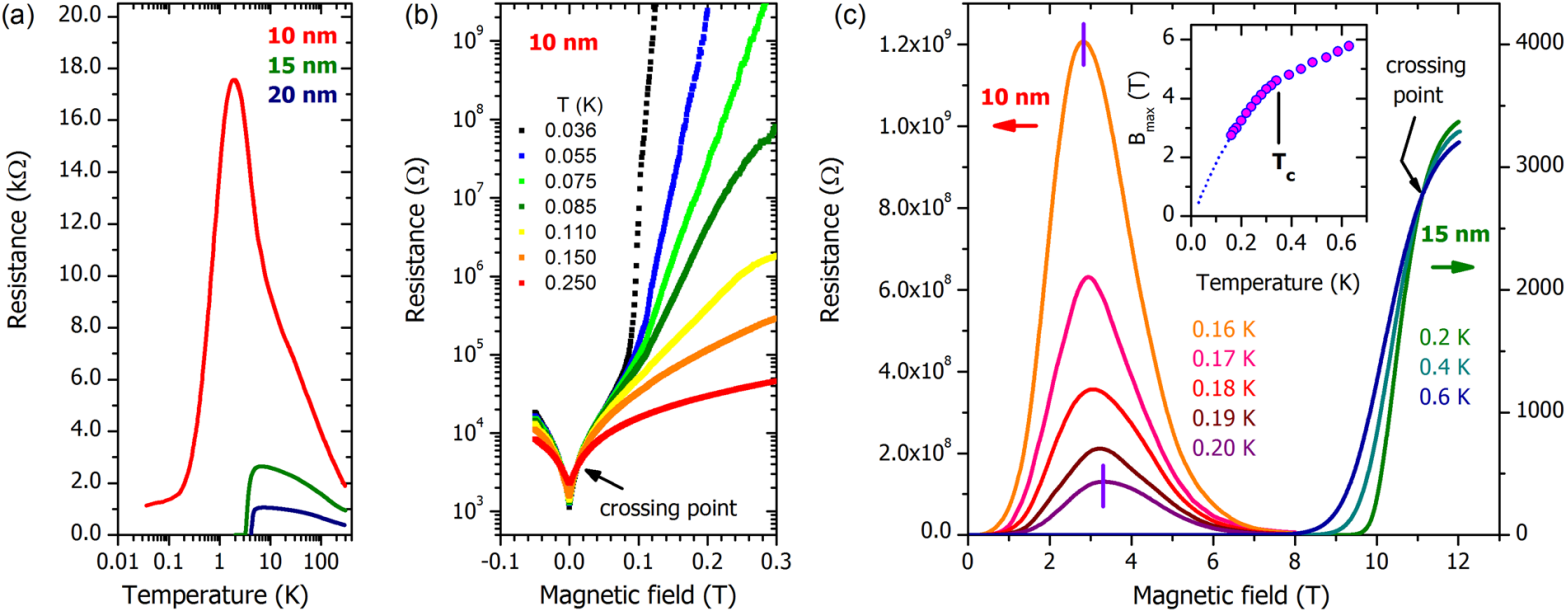
Temperature and magnetic field dependences of the resistance of NbTiN films. (**a**) The temperature dependences of the resistance in zero magnetic field for three NbTiN films of thicknesses 10, 15, and 20 nm, respectively. (**b**) Low-field isothermal magnetoresistance of the 10 nm thick film at different temperatures. At temperatures below 0.1 K, the magnetoresistance develops a sharp kink for *B* < 0.1 T, moving to lower magnetic fields as the temperature is further lowered. Above the kink, even a small increase in the field results in a sharp increase of the resistance by several orders of magnitude. The crossing point, *R*_*c*_ = 4.7 kΩ, *B*_SIT_ = 0.015 T, separates the regions with *dR*/*dT* < 0 and *dR*/*dT* > 0. (**c**) Magnetoresistance of the 10 nm and 15 nm thick films on a linear field scale. The left ordinate corresponds to the data for 10 nm thick film taken in the 0.16–0.20 K temperature range, and the right hand ordinate refers to the data in the 0.2 – 0.6 K interval for the 15 nm thick film. Note that the two resistance scales differ by a factor of 3 · 10^5^. The magnetoresistance of the 15 nm thick film exhibits the conventional superconducting behavior with a well-defined upper critical field *B*_*c*2_(0) = 10.5 T and the crossing point at *B*_*c*_ = 11 T stemming from the interplay of superconducting fluctuations contributing to conductivity^41,42^, and similar to behavior observed in materials such as InO_*x*_^43^. By contrast, the magnetoresistance of the 10 nm thick film develops a colossal insulating peak at fields well below *B*_*c*2_. The vertical strokes on the 0.16 K and 0.20 K curves for the 10 nm thick film mark the fields *B*_max_ at which the magnetoresistance peaks are achieved. The inset presents the temperature dependence of *B*_max_ (symbols). The dotted line, extrapolating the data to the *T* → 0 limit, illustrates the trend of *B*_max_ of shifting towards almost zero field upon decreasing temperature.

The films were lithographically patterned into bars and resistivity measurements were performed at sub-Kelvin temperatures in helium dilution refrigerators (see the details of the sample geometry and measurement technique in (See Supplemental Material for further information on the fabrication and measurement techniques, films structural, superconducting and transport parameters, and *I*–*V* characteristics)). The temperature dependences of the resistance, *R*(*T*), given as resistance per square, are shown in Fig. 2(a) over four decades in temperature. Upon cooling in zero magnetic field, all three films undergo a superconducting transition that manifests as a severe resistance drop. The superconducting transition temperature, *T*_*c*_, is determined by the inflection point of *R*(*T*) and marks the temperature at which thermodynamically stable Cooper pairs appear. For the most highly disordered sample, this precedes the point at which global phase coherence, and hence zero resistivity, occurs^30^. Instead, with decreasing film thickness (and increasing disorder), spatial fluctuations of the superconducting gap become increasingly important, giving rise to the formation of self-organized structures of superconducting islands in a non-superconducting environment^31,32^. The temperature *T*_*c*_ decreases with decreasing film thickness and consequent increasing sheet resistance.

## Results

The resistances of all three films exhibit peaks at temperatures just above *T*_*c*_, with the peak amplitudes increasing as the thickness decreases. Similar trends were observed in the parent compounds TiN^33^ and NbN^34^ near the SIT and were attributed to quantum contributions to conductivity due to weak localization and electron-electron interaction effects. The sheet resistance of the thinnest film achieves a maximum of 17.56 k${\rm{\Omega }}/\square$; notably, this well exceeds the quantum resistance *R*_*Q*_ = *h*/(2*e*)^2^ = 6.45 k${\rm{\Omega }}/\square$ which was widely believed to be the upper boundary for the existence of superconductivity in two dimensions^35–37^. A similar peak of 29.4 k${\rm{\Omega }}/\square$, well above *R*_*Q*_, was seen in TiN^38^.

Focusing on the behavior of the thinnest film (*d* = 10 nm), where the degree of disorder permits fully tuning the sample with experimentally-accessible magnetic fields, we note first that the global coherent superconducting state is not achieved at lowest temperatures. Instead, the behavior of the zero field resistivity suggests that the film falls into the Bose metallic state^39^, featuring a finite density of free vortices. The appearance of such vortices in the absence of a magnetic field is a fundamental feature of the BKT transition, which revolves around thermal fluctuations inducing vortex-antivortex pairs. Figure 2(b) presents a set of magnetoresistance curves, *R*(*B*), taken at different temperatures below *T*_*c*_ = 0.33 K determined by the inflection point of *R*(*T*) at zero magnetic field. All of the *R*(*B*) data presented below were measured at voltages *V* = 100 *μ*V, i.e. in the low-voltage response regime (see the discussion in the end of the “Data analysis” section below). Prominent features of these magnetoresistance curves, especially profound at lowest temperatures, are the crossing point at very low field *B*_SIT_ = 0.015 T that marks the SIT (discussed below) and the sharp kink at some temperature-dependent magnetic field above which the resistance increases extremely quickly as a function of field. The field associated with the kink shifts to lower fields with decreasing temperature. Inspecting the magnetoresistance behavior in the large field interval, one sees that it shows inherently non-monotonic behavior marked by a colossal insulating peak, see Fig. 2(c). Importantly, these peaks develop at magnetic fields for which the thicker films are still fully superconducting, i.e. the field *B*_max_, where the maximum is observed, is well below the upper critical field *B*_*c*2_. This suggests that the indefinite growth of *R*(*B*) at low temperatures/magnetic fields, as well as the peak in the resistance at higher temperatures in the 10 nm film, is an implication of Cooper pairing. The inset in Fig. 2(c) shows that the position of the maximum of the resistance peaks moves to lower fields with decreasing temperature. There is a kink in the *B*_max_(*T*) dependence at $T\simeq {T}_{c}$, with the slope decreasing significantly when passing to *T* > *T*_*c*_. Extrapolating the data to *T* → 0 shows that *B*_*max*_(*T*) shifts to nearly zero field upon decreasing the temperature. Taken together, this indicates that the mechanism that drives the system into the strongly localized state overpowers the effect of the suppression of the Cooper pairing by the magnetic field, which would be expected to diminish the resistance of the Cooper pair insulator^40^. Note here that the mere non-monotonicity of magnetoresistance cannot be taken as an indication of the SIT. For example, vortices crossing the superconducting wires under the applied current may also lead to large amplitude oscillations of the magnetoresistance.

**Figure 3:**
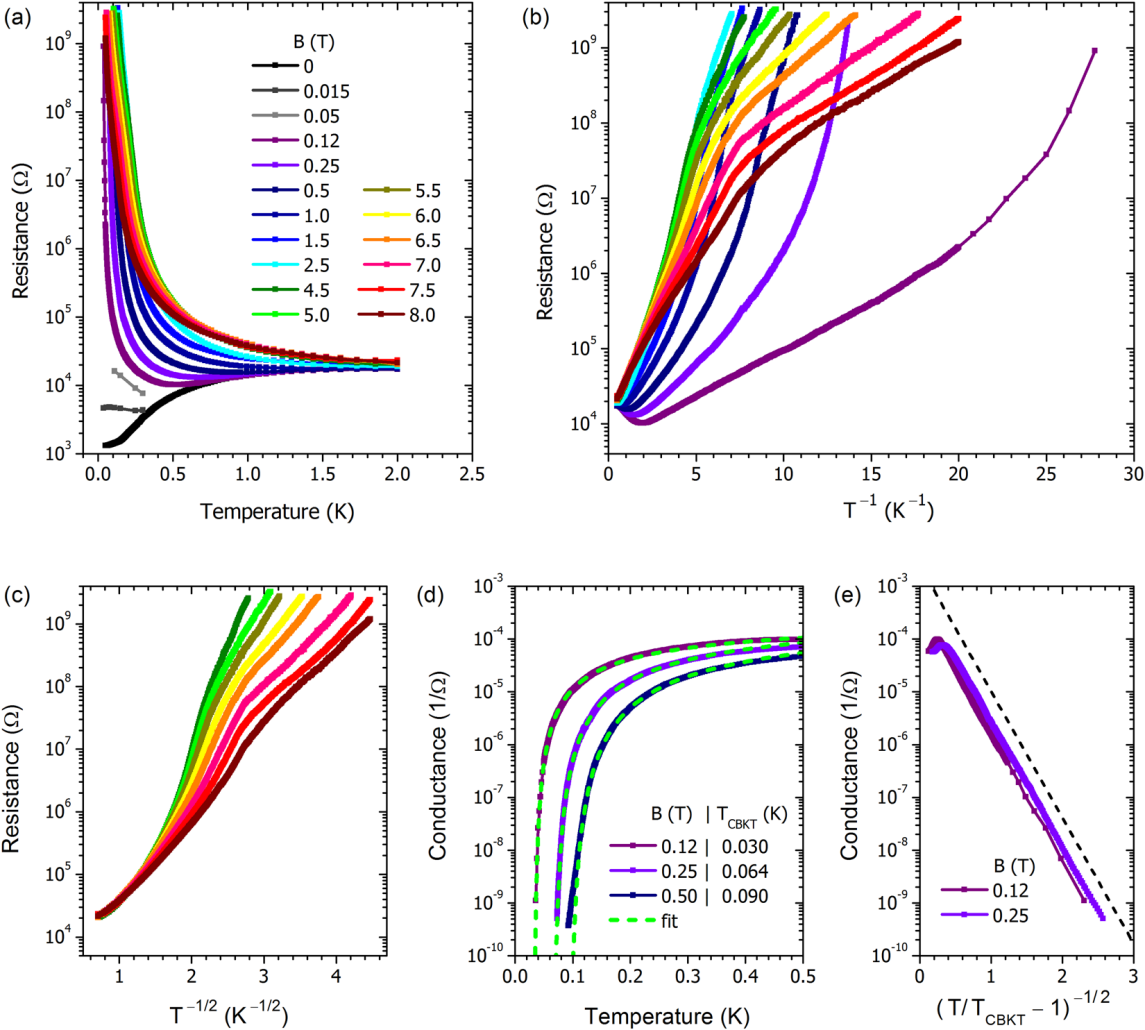
Temperature evolution of the magnetic field-induced states in the 10 nm thick NbTiN film. (**a**) Resistance vs. temperature at a series of magnetic fields listed in the legend in the panel (legend is the same for all the panels of this figure). (**b**) Resistance vs. inverse temperature at different magnetic fields. None of the temperature dependences can be modeled as Arrhenius behavior with a single activation energy. (**c**) Resistance vs. *T*^−1/2^ for magnetic fields between 4.5 and 8 T. Efros-Shklovskii behavior is likewise not observed. (**d**) Three representative curves from panel (a) replotted as conductance *G* = 1/*R*, vs. temperature, demonstrating the transition into a superinsulating state at finite temperature. The dashed lines show the best fits to Eq. (1) with the corresponding *T*_CBKT_ listed in the legend. (**e**) Conductance as a function of (*T*/*T*_CBKT_ − 1)^−1/2^ for two magnetic fields. The dashed black line is a guide to the eye revealing that linear slopes of the two curves are the same.

To gain insight into the nature of the magnetic-field-induced states, we examine *R*(*T*) at different magnetic fields. Figure 3(a) displays for the 10 nm thick film the fan-like set of magnetoresistance curves, characteristic of the magnetic field-induced SIT. The crossing point (*B*_SIT_, *R*_*c*_) in Fig. 2(b) now corresponds to nearly temperature independent *R*(*T*) at *B* = 0.015 T, separating the superconducting and insulating behavior. Two important comments are in order here. First, the field of the crossing point *B*_SIT_ = 0.015 T is *two orders of magnitude lower* than the upper critical field *B*_*c*2_. This distinguishes it from the crossing point displayed by the thicker film (*d* = 15 nm) occurring at 11 T and resulting from the quantum contributions to conductivity from superconducting fluctuations^41,42^ as well as similar crossing points in materials such as InO_*x*_^43^. Second, the resistance at the SIT is *R*_*c*_ = 4.7 kΩ, close but not equal to the quantum resistance 6.45 kΩ.

Re-plotting these data as log *R* vs. 1/*T* curves in Fig. 3(b), we see that the behavior of the resistance in the entire temperature range cannot be reduced to the Arrhenius temperature dependence with a single activation energy. Qualitatively similar behavior has been observed in thin films of the parent compound TiN^18^, as well as InO_*x*_ films^19^ and patterned Josephson Junction Arrays^20^. Instead of simple Arrhenius behavior, there is a complicated evolution of the resistance curves with increasing magnetic field. While at low fields the log *R*(1/*T*) dependence exhibits hyperactivation, i.e. faster than thermally activated growth^18^, at larger fields, the log *R*(1/*T*) curves exhibit a kink and bend down with decreasing temperature. We contrast this to the behavior of InO_*x*_ thin films^19^, where hyperactivation was likewise observed, but is consistent with an Efros-Shklovskii model followed by a crossover to a Mott hopping regime^44^. As shown in Fig. 3(c), the NbTiN film exhibits a pronounced departure from Efros-Shklovskii behavior for *B* ≤ 8 T.

In order to illuminate the physics governing the *R*(*T*) behavior, we replot the low-field data as the conductances, *G* = 1/*R*, vs. temperature in Fig. 3(d). We see in the conductance curves an insulating analogue of the drop to zero of the resistance at the onset of superconductivity. In the dual mirror picture of the conductance, we thus see the transition of the system into a superinsulating state characterized by zero conductance at finite temperature. This suggests that we can write the conductance in the generic form ln *G* ∝ −*a*/(*T* − T^*^)^*α*^ for the finite temperature zero conducting state. Using *T* ^*^ as an adjusting parameter, we find that *α* = 0.48 ± 0.03 gives the best fit to the experimental data. This is consistent with *α* = 1/2 corresponding to critical BKT behavior obtained by a detailed renormalization group analysis^45^ of the system with infinite screening length:

1 $$G\propto \exp \,(-\tfrac{b}{\sqrt{(T/{T}_{{\rm{CBKT}}})-1}}),$$

where *T*_CBKT_ replaces T^*^, and *b* is a constant of order unity. In Fig. 3(e) we plot *G* vs. (*T*/*T*_CBKT_ − 1)^−1/2^ for fields 0.12 and 0.25 T. The correct choice of the only adjustable parameter, *T*_CBKT_, for each field (shown in the legend for Fig. 3(d)), produces a linear dependence, allowing the determination of *b* as the slope of the respective lines. The dashed lines in Fig. 3(d) correspond to Eq. (1). A close inspection of the fits reveals that while the conductance curves at *B* = 0.12 and 0.25 T precisely follow the formula over six decades, the 0.50 T curve displays a slight departure (Fig. 3(d)), indicating that the assumption of critical CBKT behavior combined with an infinite screening length has started to break down.

These results establish a superinsulator as a confined low temperature charge BKT phase of the Cooper pair insulator. In this phase, vortices form a Bose condensate that completely blocks the motion of the Cooper pairs.

## Data analysis

**Figure 4:**
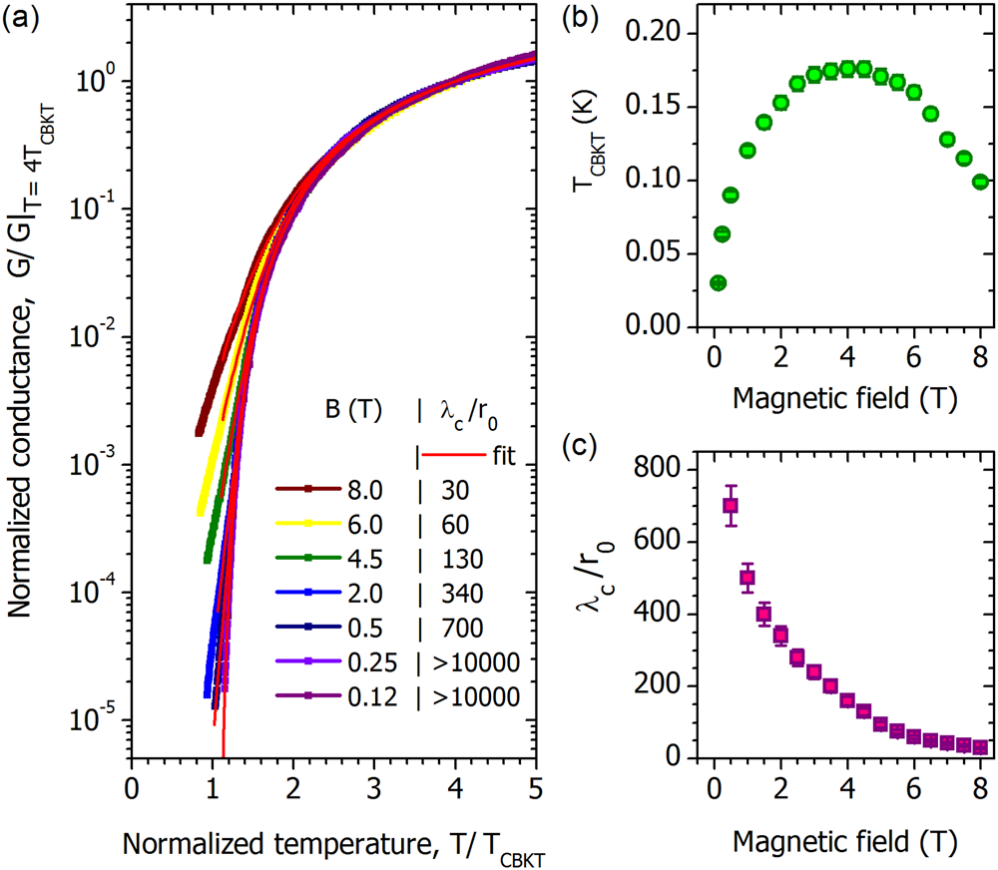
Charge BKT in the 10 nm NbTiN film. (**a**) Normalized conductance vs. normalized temperature. The temperature is normalized with respect to *T*_CBKT_ and the conductances were normalized by their values at high temperatures, *T* = 4*T*_CBKT_. Symbols stand for experimental data, and red lines show the fit obtained from the self-consistent solution to Eqs (2) and (3). We present data covering the full range of magnetic field but omit a few curves to avoid overcrowding the plot. (**b**) Magnetic field dependence of the transition temperatures *T*_CBKT_. (**c**) The screening length *λ*_*c*_ in units of *r*_0_ vs. magnetic field.

To proceed further with the analysis, we choose the value of *T*_CBKT_ for every isomagnetic *G*(*T*) curve and plot in Fig. 4(a) the conductance normalized by its value at temperature *T* = 4*T*_CBKT_ vs. the normalized temperature *T*/*T*_CBKT_(*B*). The corresponding field-dependent charge BKT transition temperature *T*_CBKT_ is shown in Fig. 4(b). Remarkably, the complex diversity of the R(T) curves (exhibiting sub-activation at high magnetic field and hyper-activation at small field in Fig. 3(b)) rescaled onto a set of uniform curves that collapse at *T*/*T*_CBKT_ > 2.5. Moreover, the field-dependent evolution of the curve shapes, including the change in concavity, now reduces simply to a successive deviation from the universal curve: the higher the magnetic field at which the curve is measured, the higher the *T*/*T*_CBKT_ ratio at which the given curve departs from the universal envelope. We stress that the above normalization procedure does not presume any special temperature dependence of *G*(*T*). The choice of the temperature at which the normalizing value of the conductance is taken is somewhat arbitrary, as seen from the quality of the collapse over the 2.5–5 range in *T*/*T*_CBKT_.

We now describe the overall *G*(*T*, *B*) behavior using a two dimensional Coulomb gas model^5^ that generalizes the Kosterlitz formula (1) by incorporating a self-consistent solution to the effects of electronic screening. The conduction is controlled by the density of free charge carriers, *G* ∝ *n*_*f*_, i.e. the conductance is proportional to the inverse squared mean distance between the carriers. In the critical BKT region, *n*_*f*_ is the 2D density of the unbound charges, which is related to the correlation length *λ* at which the unbound charges appear via the equation:

2 $${n}_{f}=\frac{1}{2\pi }(\frac{1}{{\lambda }^{2}}-\frac{1}{{\lambda }_{c}^{2}}),$$

where *λ*_*c*_ is the smaller of the bare electrostatic screening length of the film, Λ, or the lateral linear dimension of the film. The screening length defines the maximal spatial scale of logarithmic charge interactions in the film, *V*(*r*) ∝ ln(*r*/Λ) for *r*_0_ < *r* < Λ. Since ${r}_{0}\approx d\ll {\rm{\Lambda }}$, the film is effectively 2D with respect to the Coulomb charge interactions over a broad range of length scales.

Relating *λ*_*c*_ and the density of the unbound charges through the Poisson equation, we derive, following^5^, a self-consistent equation for *λ*:

3 $${(\frac{\lambda }{{r}_{0}})}^{\sqrt{(T/{T}_{{\rm{CBKT}}})-1}}={{-}\kern-0.5em Z}\cdot (1-\frac{{\lambda }^{2}}{{\lambda }_{c}^{2}}),$$

where ${{-}\kern-0.5em Z}$ is a constant. As *λ*_*c*_ → ∞, Eqs (2) and (3), reduce to Eq. (1), with *b* = 2 ln(1/${{-}\kern-0.5em Z}$).

We verify this model against the data by fitting a self-consistent solution of Eqs (2) and (3) to the measured isomagnetic G(T) (see representative curves in Fig. 4(a)); the only adjustable parameter in these fits is the screening length *λ*_*c*_. The field evolution of the derived *λ*_*c*_ values is shown in Fig. 4(c). Thus, we see that the two-dimensional Coulomb gas model fully describes the complex diversity of the experimental data, including both the BKT critical behavior and the deviation from criticality, using only one adjustable parameter.

**Figure 5:**
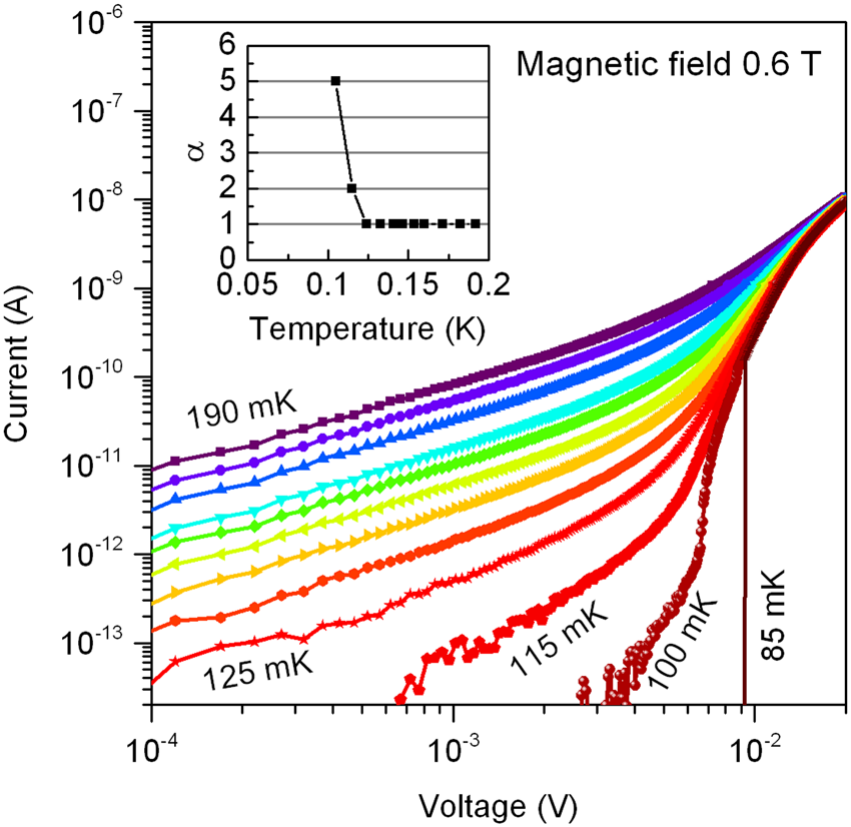
Current-voltage curves in the vicinity of the charge BKT transition. Main panels: Current-voltage curves for a range of temperatures at *B* = 0.6 T. Inset: Onset of superlinear behavior below *T*_CBKT_, obtained by fitting the low-current portion of the *I*-*V* curves to *I* ∝ *V*^*α*^.

A signature of the BKT transition is a current-voltage curve of the form *I* ∝ *V*^*α*^ at *T* ≤ *T*_BKT_, with *α* experiencing a jump from *α* = 1 to *α* = 3 at *T* = *T*_BKT_. For our NbTiN films, Fig. 5 displays a set of *I*–*V* curves measured as a function of temperature for magnetic field *B* = 0.6 T (*I*–*V* curves for additional fields are shown in (See Supplemental Material for further information on the fabrication and measurement techniques, films structural, superconducting and transport parameters, and *I*–*V* characteristics)). In this field, the electrostatic screening length is approximately equal to the lateral linear size of the film (see Fig. 4(c)). *T*_BKT_ determined from *I*(*V*) coincides with *T*_BKT_ determined from *R*(*T*).

## Discussion and Conclusion

**Figure 6:**
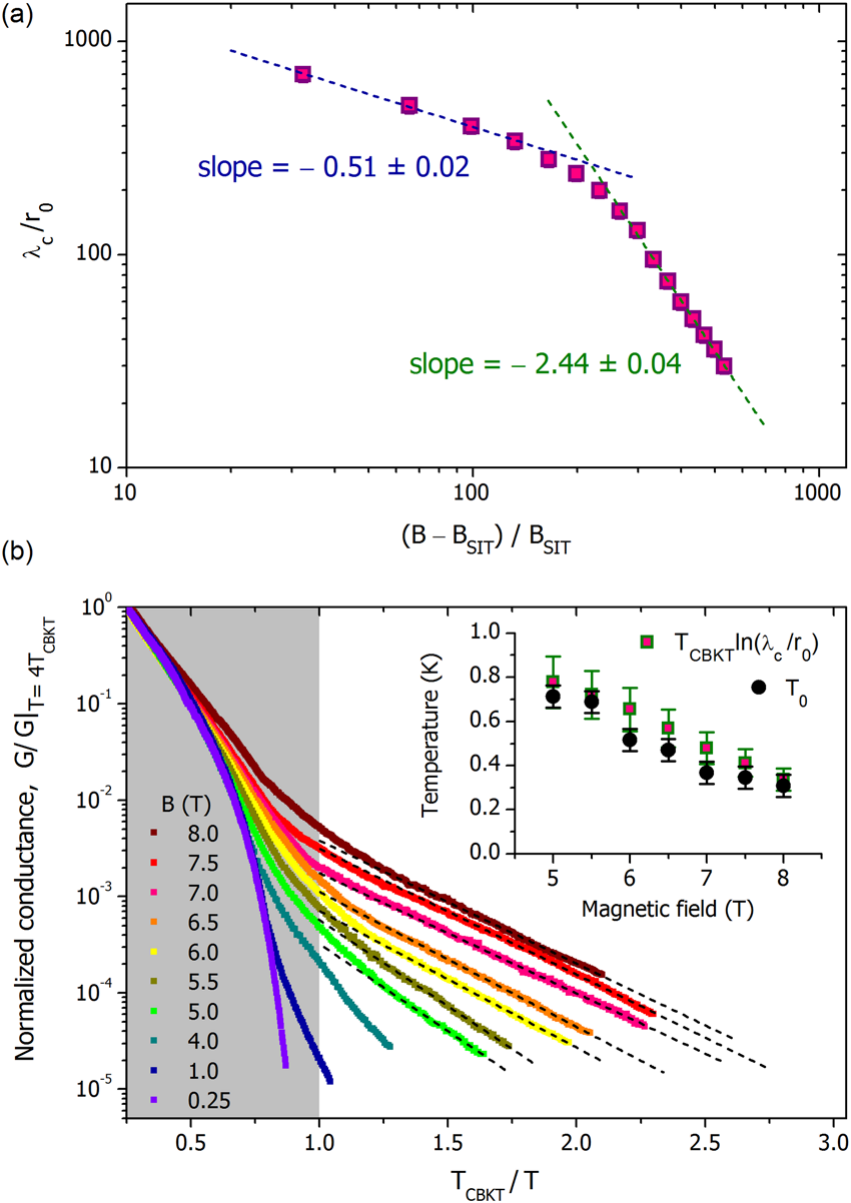
Critical behavior and the effects of the finite electrostatic screening length. (**a**) The screening length *λ*_*c*_ in units of *r*_0_ in a double-log scale as function of (*B* − *B*_SIT_)/*B*_SIT_. At low magnetic fields *λ*_*c*_ follows the power-law dependence shown by the dashed line, *λ*_*c*_ ∝ (*B* − *B*_SIT_)^−*ν*^, with *ν* = 0.51 ± 0.02. At higher magnetic fields the slope increases and the exponent becomes *ν*′ = 2.44 ± 0.04. (**b**) Normalized Arrhenius plots of conductances at various fields. The shaded area corresponds to the critical region above *T*_CBKT_ described by Eqs (2) and (3). The tails at *T* < *T*_CBKT_ demonstrate thermally activated behavior highlighted by the dashed lines. The slopes grow with decreasing magnetic field, the corresponding values *T*_0_ of the activation temperature are shown in the inset as black circles. The inset presents a comparison of the activation energies, *T*_0_, and *T*_CBKT_ ln(*λ*_*c*_/*r*_0_) shown by squares, evidencing the logarithmic dependence of the activation energy on the screening length, as expected for the 2D logarithmic Coulomb interaction between the charges.

We now discuss the implications of identifying the nature of the Cooper pair insulator as a two-dimensional neutral Coulomb plasma of excessive/deficit Cooper pairs, each carrying the charge ±2*e*, and analyze further the parameters of this Cooper pair plasma. We note first the apparent divergence of *λ*_*c*_ upon decreasing *B*. That *λ*_*c*_ depends on *B* enables its identification as the electrostatic screening length in disordered films, *λ*_*c*_ = Λ = *εd*. Accordingly, its divergence upon decreasing *B* corresponds to the divergence of the dielectric constant upon approach to the SIT, with a cutoff of the divergence as *λ*_*c*_ approaches the lateral system size. The value of the magnetic field, *B*_SIT_ = 0.015 T, where the SIT occurs, is determined by the crossing point of the resistive curves, as seen in Fig. 2(b), whose appearance is a hallmark of the field-driven SIT. In the vicinity of the field-driven SIT the corresponding correlation length diverges as ${(B-{B}_{{\rm{SIT}}})}^{-\tilde{\nu }}$^35^, as do the relevant physical quantities. To analyze the character of the divergence we plot *λ*_*c*_/*r*_0_ vs (*B* − *B*_SIT_)/*B*_SIT_ (Fig. 6(a)), where we see that at the lowest fields *λ*_*c*_ ∝ (*B* − *B*_SIT_)^−*ν*^, with *ν* = 0.51 ± 0.02 and *ν* = 2.44 ± 0.04 further from the transition. In strongly disordered films, one can expect the SIT to be governed by a percolation transition (see^11^ and references therein), so it is natural to analyze the scaling behavior in the framework of a random JJA or a random inductor-capacitor network (ICN), with the magnetic field moving the system away from the percolation transition at *B* = *B*_SIT_. At large field, the screening length *λ*_*c*_ is not large, and hence the electric behavior of the network is dominated by the nodes’ capacitances to ground *C*_0_^11,40^. Numerical analysis of the ICN in the vicinity of the transition in the large *C*_0_ limit^46^ yields *ν* = 2.52, in good agreement with our experimental findings. This establishes the percolative behavior near the transition and suggests that the magnetic field indeed acts as the parameter destroying the superconducting bonds on approach to the SIT from the superconducting side. We thus expect that the superfluid density should scale as *ρ*_*s*_ ∝ (*B*_SIT_ − *B*)^*ν*^. In the limit of *C*_0_ → 0, i.e. in the region of large *λ*_*c*_^40^, the duality principle^11,35,36^ implies that on the insulating side the dielectric constant scales as $\varepsilon \sim {\rho }_{s}^{-1}$. The exponent *ν* = 0.53 in the dual limit was found in^47^, in agreement with our experimental result of *ν* = 0.51 ± 0.02.

Our finding is in a full accord with the polarization catastrophe paradigm built on the divergence of *ε* as function of the carrier concentration at the approach to the quantum metal-insulator transition^48,49^. In our experiment, it is *B* − *B*_SIT_ that plays the role of the deviation of the carrier concentration from its critical value.

Having determined the screening length and *T*_CBKT_ as a function of magnetic field, we now can make an independent cross-check on the 2D Coulomb nature of the superinsulator. Shown in Fig. 6(b) are Arrhenius plots of the normalized conductance vs. *T*_CBKT_/*T* at various magnetic fields, highlighting the thermally activated behavior at low temperatures, *T* < *T*_CBKT_. Note that Eqs (2) and (3) describe conductance only at *T* > *T*_CBKT_, shown as a shaded rectangle. The field dependent activation temperatures *T*_0_ are presented in the inset to Fig. 6(b). When the typical size of the unbound pair becomes comparable to Λ, the interaction ceases to be logarithmic and the conductance is dominated by thermodynamically activated free charges. The onset of this activated behavior marks the point at which the correlation length becomes comparable to the screening length. Thus, the low-temperature tails in *G*(*T*) are expected to be exponential and to depart from the BKT criticality curve. Theoretical calculations^10,40^ and simulations^50^ of thermally activated hopping transport in a 2D insulator with logarithmic Coulomb interactions between the charge carriers yield an activation temperature ${T}_{0}\simeq {T}_{{\rm{CBKT}}}\,\mathrm{ln}({\lambda }_{c}/{r}_{0})$. In the same inset we present our experimental values of *T*_CBKT_ ln(*λ*_*c*_/*r*_0_) at the same fields; these indeed appear remarkably close to the independently determined *T*_0_ in accord with the theoretical expectations. This correspondence validates the 2D Coulomb logarithmic interaction between charges at distances not exceeding the screening length. Similarly, exponential low-temperature tails in the resistance were observed in JJA on the superconducting side of the SIT. The tails appeared below the vortex BKT transition temperature where the applied magnetic field introduced the excess unbound vortices^51^.

We now can resolve the long-standing open question in the study of the SIT: the origin of the giant peak in the magnetoresistance^17,19,52–54^. It arises from the combination of the dielectric constant rapidly decaying with the increase of the magnetic field and the nonmonotonic behavior of *T*_CBKT_. In order to gain insight into the behavior of the latter, we employ the model of JJA, an array of superconducting granules connected with Josephson links, which is a long-standing model for a critically disordered superconducting film^55^. In the parent compound NbN, for example, disordered films were seen to have a self-induced granular structure with the elemental cell having characteristic size ~1–2$\xi$^56^. It also should be noted that the relevant disorder is electronic rather than structural, and can emerge from structurally homogenous films, particularly when in close proximity to a SIT^57^. The origin of the nonmonotonic behavior of *T*_CBKT_ can be explained by recalling that the energy gap of the Cooper pair insulator in JJA, Δ_*c*_(*B*), is suppressed by the Josephson coupling *E*_*J*_ between the neighboring granules, Δ_*c*_(*B*) = Δ_*c*_(0)[1 − *AE*_J_(*B*)/*E*_*c*_]^40^, where *E*_*c*_ is the Coulomb energy of a single granule and *A* is a constant. The Josephson coupling is maximal at zero field and, in the irregular JJA, has the minimum at the frustration factor *f* = 1/2^58^, where *f* ≡ *BS*/Φ_0_, *S* is the average area of the JJA elemental cell and Φ_0_ is the magnetic flux quantum. Accordingly, the effective Coulomb energy acquires the *maximum* at *f* = 1/2, i.e. the nonmonotonic behavior of *T*_CBKT_ reflects the nonmonotonic behavior of *E*_J_ as a function of the magnetic field. The non-monotonic nature of *E*_J_ also can be directly observed in *I*–*V* curves, where the activation voltage varies non-monotonically with field (see SI). This enables us to estimate the parameters of the system as follows. The observed maximum in *T*_CBKT_ at *B* ≈ 4 T (Fig. 4(b)) implies that the average area of an elemental cell of our self-induced granular structure, *S* ≈ 260 nm^2^ and, hence, the linear size of the elemental cell $\sqrt{S}\approx 16$ nm ≈ 3.5*ξ*, where *ξ* = 4.5 nm is the superconducting coherence length of the NbTiN film (see SI). Interestingly, this correlates with the analogous estimates for TiN, where $\sqrt{S}\approx 4\xi$ was observed^59^. The described nonmonotonic behavior is accompanied by the overall suppression of the superconducting gap by the increasing magnetic field. The latter eventually would suppress the superconducting gap in Cooper pair droplets and hence Δ_*c*_, resulting in a further drop of the resistance. Then, the Cooper pair insulator ends up as a metal^38^.

By comparison to NbTiN, the behaviors complying with the formation of the superinsulating state were observed in other materials at very low temperatures. In TiN films the superinsulator appeared at 40 mK^24^. More recently, the finite temperature zero-conductance state in InO_*x*_ was reported at $T\mathop{ < }\limits_{ \tilde {}}35$ mK^19^. The temperature dependence of the conductance in InO_*x*_ was found to follow the so-called Vogel-Fulcher-Tamman dependence, *σ* ∝ exp[−const/(T^*^ − *T*)]^60–62^. This, however, can be viewed as a manifestation of the same BKT physics, but in a more disordered system^63^.

The Kosterlitz-like exponential behavior described by Eq. (1) also appears in the framework of many-body localization (MBL) theory^64,65^. One can thus ask if the observed critical behavior of the NbTiN films should instead be considered in the context of MBL. A detailed comparison of BKT and MBL physics is given in^63^, but we briefly note that our data and analysis unambiguously evidences the primary role of the long range two dimensional logarithmic Coulomb interactions between charges which are a platform for BKT physics. By contrast, the original MBL model^64,65^ was constructed for one dimensional systems in the strict absence of the long range interactions. While recent work has proposed the extension of MBL to a model with long range interactions^66^, it is not clear whether the exponential critical behavior would survive in the systems with interactions. Even more important is that in the MBL framework, taking the system deeper into the insulator (i.e. moving away from the SIT) via increasing magnetic field and/or disorder is expected to enhance localization and hence critical behavior. As discussed above, we observe the opposite trend: increasing magnetic field shrinks the screening length and narrows the window in which the criticality is seen, consistent with the expectations of a BKT system. In sum, this indicates that at present MBL does not offer the appropriate framework for describing the superinsulating state and its related criticality.

To summarize, our findings clearly establish the finite temperature superinsulating state in NbTiN as the low temperature charge BKT phase of the Cooper pair insulator. We demonstrate superinsulating behavior in a new material with a substantially higher transition temperature of nearly 200 mK, allowing for the first time a detailed characterization of behavior of the system both above and below *T*_CBKT_ and its evolution in a wide range of magnetic fields.

## Methods

The fabrication is built upon the Atomic Layer Deposition technique. The structure of films grown on Si substrates with AlN buffer layers was investigated using a JEOL-4000EX electron microscope operated at 400 kV, with a point-to-point resolution of 0.16 nm and a line resolution of 0.1 nm. The details of sample fabrication, analysis and measurements are given in SM.

### Data availability

The authors declare that all relevant data supporting the findings of this study are available within the article and its supplementary information file.

## Electronic supplementary material

Supplementary Materials
