# Supplementary material for: Charge Berezinskii-Kosterlitz-Thouless transition in superconducting NbTiN films
Source: Sci Rep. 2018 Mar 6;8:4082. doi: 10.1038/s41598-018-22451-1 (PMC5840303; doi:10.1038/s41598-018-22451-1)
Supplement: Supplementary file 1 — Supplementary Materials [file 41598_2018_22451_MOESM1_ESM.pdf]

Supplementary Materials

Charge Berezinskii-Kosterlitz-Thouless transition in superconducting NbTiN films

Alexey Yu. Mironov,[1,2,3] Daniel M. Silevitch,[4] Thomas Proslier,[5] Svetlana V. Postolova,[1,2] Maria V. Burdastyh,[1,2] Anton K. Gutakovskii,[1,2] Thomas F. Rosenbaum,[4] Valerii M. Vinokur,[6,7] and Tatyana I. Baturina[1,2,3,8]

[1]*A. V. Rzhanov Institute of Semiconductor Physics SB RAS, 13 Lavrentjev Avenue, Novosibirsk 630090, Russia*
[2]*Novosibirsk State University, Pirogova str. 2, Novosibirsk 630090, Russia*
[3]*The James Franck Institute and Department of Physics, The University of Chicago, Chicago, Illinois 60637, USA*
[4]*Division of Physics, Mathematics, and Astronomy, California Institute of Technology, Pasadena, California 91125, USA*
[5]*Institut de recherches sur les lois fundamentales de l'univers, Commissariat de l'énergie atomique et aux énergies renouvelables-Saclay, Gif-sur-Yvette, France*
[6]*Materials Science Division, Argonne National Laboratory, Argonne, Illinois 60439, USA*
[7]*Computation Institute, University of Chicago, 5735 S. Ellis Avenue, Chicago, IL 60637, USA*
[8]*Departamento de Física de la Materia Condensada, Instituto de Ciencia de Materiales Nicolás Cabrera and Condensed Matter Physics Center (IFIMAC), Universidad Autónoma de Madrid, 28049 Madrid, Spain*

Fabrication technique

The Atomic Layer Deposition (ALD) growths were carried out in a custom-made viscous flow ALD reactor in the self limiting regime. A constant flow of ultrahigh-purity nitrogen (UHP, 99.999%, Airgas) at $\sim$ 350 sccm with a pressure of $\sim$ 1.1 Torr was maintained by mass flow controllers. An inert gas purifier (Entegris GateKeeper) was used to further purify the N_2 gas by reducing the contamination level of H_2, CO, and CO_2 to less than 1 ppb and O_2 and H_2O to less than 100 ppt. The thermal ALD growth of the AlN/NbTiN multilayer was performed using alternating exposures to the following gaseous reactants with the corresponding timing sequence (exposure-purge) in seconds: $AlCl_3$ (anhydrous, 99.999%, Sigma-Aldrich) (1 - 10), $NbCl_5$ (anhydrous, 99.995%, Sigma-Aldrich) (1 - 10), $TiCl_4$ (99.995%, Sigma-Aldrich) (0.5 - 10) and NH_3 (anhydrous, 99.9995%, Sigma-Aldrich) (1.5 - 10). The intrinsic silicon substrates were initially cleaned in-situ using a 60 s exposure to O_3 repeated 5 times. The AlN buffer layer of thickness 7.5±0.5 nm was deposited at 450° C with 200 ALD cycles. The chamber temperature was then lowered to 350° C to synthesize the NbTiN layers. The growth cycle of the NbTiN is 2×($TiCl_4$ + NH_3) and 1×($NbCl_5$ + NH_3) that was repeated 80, 140, and 256 times with the corresponding total ALD cycles 240, 420 and 768 to produce the different film thicknesses 10 nm, 15 nm, and 20 nm, respectively as measured ex-situ by X-ray reflectivity (XRR). The chemical composition measured by X-ray Photoemission Spectroscopy (XPS) and Rutherford Backscattering Spectroscopy (RBS) show consistently for the AlN layer 5.5±0.3% of Cl impurities and a Al/N ratio of 1±0.05, whereas for the NbTiN films 3±0.3% of Cl impurities, a Nb/Ti ratio of 2.3±0.03 and a (Nb+Ti)/N ratio of 1±0.03. The material densities measured by RBS and XRR are 2.5±0.01 g/cm^3 in AlN and 6±0.05 g/cm^3 in the NbTiN.

Analysis of structure and composition of NbTiN films

The structure of $Nb_xTi_{1-x}N$ films grown on Si substrates with AlN buffer layers was investigated using a JEOL-4000EX electron microscope operated at 400 kV, with a point-to-point resolution of 0.16 nm and a line resolution of 0.1 nm. The Digital Micrograph software (GATAN) was used for the digital processing of High Resolution Transmission Electron Microscopy (HRTEM) images. The interplanar spacings were determined to an accuracy of 0.05 nm. The images displayed in Figs. 1(a,b) were calibrated using the lattice of the crystalline Si substrate clearly visible in the HRTEM image presented in Fig. 1(a). In order to determine structure and composition of the NbTiN films, we use electron diffraction data (Fig. 1(c)) which contain characteristic rings and point reflexes. The latter were identified as belonging to the (220) order of the Si substrate with interplanar spacing $d = 1.92$ Å and were used as a reference scale for determining the interplanar spacing of the NbTiN film. The detailed analyses of the electron-diffraction data reveals that our NbTiN films have the same rock-salt crystalline structure as both NbN and TiN. We find that the

lattice constant of the NbTiN film is $a = 4.33$ Å. The values of the interplanar spacings corresponding to the different planes are derived from the positions of the ring brightness maxima shown in Fig. 1(d) and are presented in Table S I. The AlN buffer layer has hexagonal lattice 63 mc; the (100) order of the buffer layer has $d = 2.7$ Å. The composition of the NbTiN films is found using Vegard's law,

$$a_{\rm NbTiN} = x \cdot a_{\rm NbN} + (1-x)\cdot a_{\rm TiN}, \quad \text{where } a \quad \text{is the lattice constant and } 0 \leq x \leq 1\,. \tag{1}$$

Comparison with the tabulated data on NbN and TiN (see Table S I) yields that NbTiN is a solid solution of NbN [1, 2] and TiN [1, 3] with $x = 0.7 \pm 0.02$.

Table S I: Lattice Parameters. The tabulated parameters for NbN and TiN, and experimental values for NbTiN. d is the interplane distance.

	NbN	TiN	NbTiN
lattice constant	4.39 [Å]	4.24 [Å]	4.33 [Å]
plane	d [Å]	d [Å]	d [Å]
(111)	2.54	2.45	2.51
(200)	2.20	2.12	2.17
(220)	1.55	1.5	1.54
(311)	1.32	1.28	1.31
(222)	1.27	1.22	1.25
(420)	0.98	0.95	0.97
(422)	0.90	0.87	0.89

Samples and Measurements

To carry out transport measurements, NbTiN films were patterned using photolithography and plasma etching into 10-contact resistivity bars 50 μm wide and with 100, 250, and 100 μm separation between the voltage probes (Fig. S1). The chosen design allows for Hall effect measurements and both two-probe and four-probe resistivity measurements.

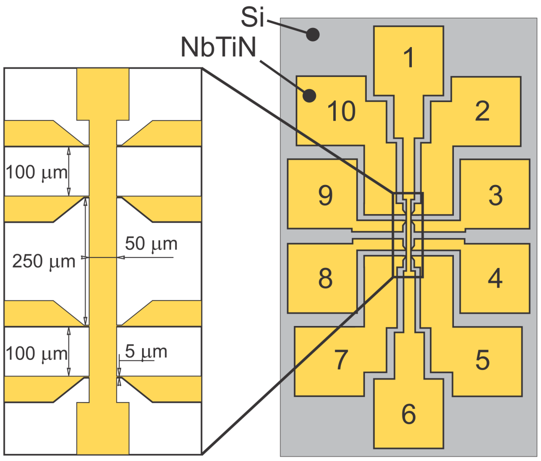

Figure S 1: Schematic of lithographically-patterned sample configuration

Measurements of the temperature and magnetic field dependences of the resistance were carried out in helium dilution refrigerators. The magnetic field was always perpendicular to the plane of the sample. In the low resistance

range, standard four-probe constant-current measurements were performed at 10 nA and 3 Hz. In the high resistance range, a two-probe constant-voltage technique was used instead, with a 100 μV/3 Hz probe. The excitations in both cases were verified to be in the linear response regime. Both sets of measurements were performed using SR830 lock-in amplifiers. The high-resistivity measurements also employed an SR570 low-noise current preamplifier. The resistance per square in the two-probe geometry was determined by matching the two-probe and four probe measurements at high temperature ($T \sim 10$ K).

Table S II: Parameters of NbTiN films:
d is the film thickness, T_c is the superconducting critical temperature defined by the inflection point of $R(T)$, R_{300} is the room temperature resistance, $B_{c2}(0)$ is the upper critical field, D is the diffusion constant, $\xi_d(0)$ is the superconducting coherence length.

d [nm]	T_c [K]	R_{300}	$B_{c2}(0)$ [T]	D [cm^2/c]	$\xi_d(0)$ [nm]
10	0.33	1900	—	—	—
15	3.35	940	10.5	0.24	4.65
20	4.27	390	12	0.27	4.35

Measurements of the Hall effect in 10 nm thick film yields the carrier density $n = 4.7 \cdot 10^{21}\,\text{cm}^{-3}$. The upper critical field $B_{c2}(0)$ for the 15 and 20 nm films was estimated as $B_c/1.05$, where B_c is the crossing point [4, 5]. The crossing point for the 15 nm thick film is shown in Fig. 2(c). The diffusion coefficient, D, and the superconducting coherence length, $\xi_d(0)$, are given by

$$D = \frac{\pi k_{\rm B} T_c}{2\gamma e B_{c2}(0)}, \qquad (2)$$

$$\xi_d(0) = 0.85\sqrt{\xi_0 l} = 0.85\sqrt{\frac{3}{2\pi}\frac{\hbar}{eB_{c2}(0)}}\,, \qquad (3)$$

where $k_{\rm B}$ is Boltzmann's constant, γ is Euler's constant, and $\gamma \approx 1.781$.

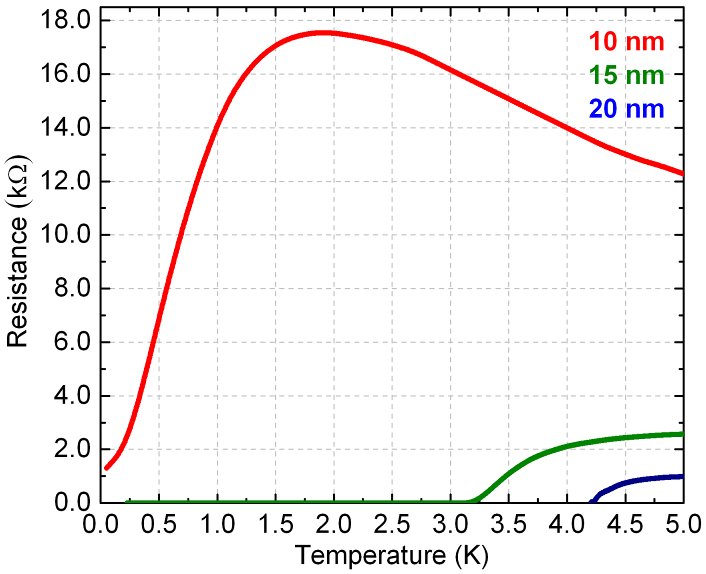

Figure S 2: Temperature dependences of the resistance in zero magnetic field for three NbTiN films of thicknesses 10, 15, and 20 nm respectively.

On the possibility of observation BKT-transition from $I - V$ measurements

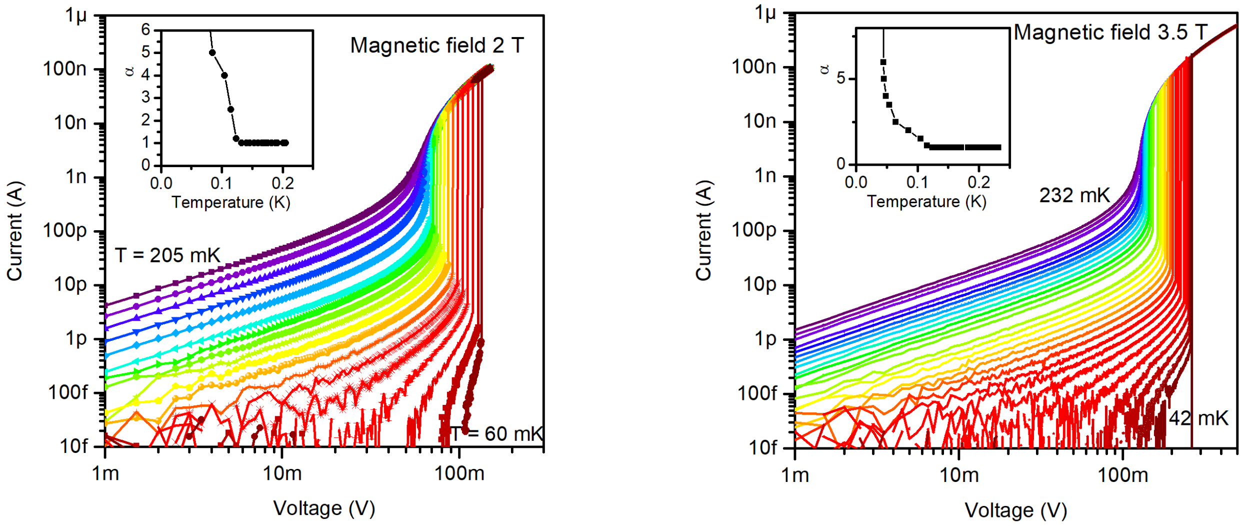

Figure S 3: Current-voltage characteristics for film $d = 10\,\mathrm{nm}$ measured at different temperatures in magnetic field: (a) $B = 2\,\mathrm{T}$; (b) $B = 3.5\,\mathrm{T}$. Insets: Temperature dependance of α extracted from $I \propto V^{\alpha}$ at low voltage.

Current-voltage curves of the form $I \sim V^{\alpha}$ are a phenomenological signature of the BKT transition, with α experiencing a jump from $\alpha = 1$ to $\alpha = 3$ at $T = T_{\mathrm{BKT}}$, and then behaving as $\alpha = 1 + 2(T_{\mathrm{BKT}}/T)$ for $T < T_{\mathrm{BKT}}$. While it is often believed to be the most reliable test for identifying the BKT transition, there are issues which need to be understood lest they mask the expected BKT behavior, for both the charge and vortex BKT transitions. First, $I - V$ measurements at $T < T_{\mathrm{BKT}}$ are necessarily taken in a highly nonlinear regime. Due to the effects of Ohmic heating, the pure BKT power law is expected to hold only in the low-current regime, and the high current limit of the fit ranges needs to be restricted to minimize the impact of heating. In addition, at sufficiently small drive, the BKT physics becomes cut off as the size of the dissociating BKT dipole becomes comparable to the size of the sample. For the charge BKT transition in particular, the screening length in the insulating phase (typically of order ten elemental units for Josephson Junction Arrays) further limits the maximum effective size. Thus, the window within which $I \sim V^{\alpha}$ is a reliable probe of BKT physics is restricted from both sides. The systems which maximize the width of this window and hence offer the cleanest realizations of the BKT $I - V$ behavior are thin strongly disordered superconducting films close to the SIT to ensure a sufficiently large screening length. An example of an observation of a real jump in $\alpha(T)$ is given in Ref. [6]. More common, however, is the result seen in Ref. [7] where $\alpha(T)$ shows a smooth temperature dependence with no discontinuous jump at the transition.

In NbTiN, we see both situations depending on the magnetic field. For $B = 0.6$ T (main text Fig. 5), the electrostatic screening length is comparable to the lateral size of the film (main text Fig. 4(c)), and as a result the onset temperature and shape of the $I - V$ curves are consistent with the behavior determined from the $R(T)$ curves. By contrast, at higher fields (Fig. S3) the electrostatic screening lengths are substantially smaller than the film size and hence there is an suppression of seeming T_{BKT} in the $I - V$ measurements as compared those inferred from $R(T)$: by the factor 1.5 for the field $B = 2\,\mathrm{T}$ and by factor of 2 for $B = 3.5$T.

On the nonmonotonic behavior of $T_{\mathrm{BKT}}(B)$

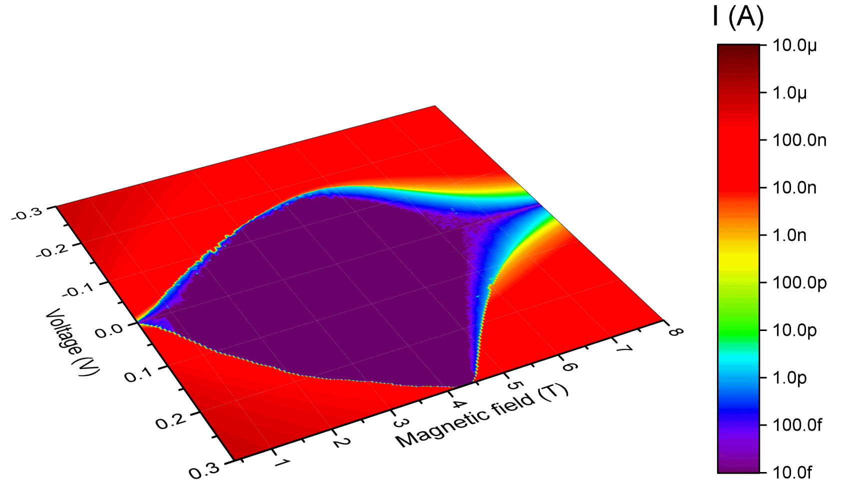

Figure S 4: I versus B and V at $T = 25$ mK. The border enclosing the infinitely resistive superinsulating domain (in violet) delineates the dependence of the threshold voltage upon magnetic field. The observed non-monotonic dependence of threshold voltage of $I(V)$ on magnetic field is a reflection of the non-monotonic dependence of the Josephson energy on magnetic field. The threshold voltage exhibits a maximum at $B \simeq 4.3\,\mathrm{T}$ consistent with the behavior of $T_{\mathrm{BKT}}(B)$.

SUPPLEMENTARY REFERENCES

[1] H. O. Pierson, *Handbook of refractory carbides and nitrides: properties, characteristics, processing and applications* (Noyes publications, Westwood, New Jersey, 1996).

[2] A. V. Lindea, R. M. Marin-Ayral, F. Bosc-Rouessac, and V. V. Grachev, *International Journal of Self-Propagating HighTemperature Synthesis* **19**, 9 (2010).

[3] V. Valvoda, *Journal of Alloys and Compounds* **219**, 83 (1995).

[4] T. I. Baturina, J. Bentner, C. Strunk, M. R. Baklanov, A. Satta, *From quantum corrections to magnetic-field-tuned superconductor-insulator quantum phase transition in TiN film*, Physica B **359-361**, 500 (2005).

[5] K. S. Tikhonov, G. Schwiete, and A. M. Finkel'stein, *Fluctuation conductivity in disordered superconducting films*, Phys. Rev. B**85**, 174527 (2012).

[6] P. G. Baity, X. Shi, Z. Shi, L. Benfatto, and D. Popovic. *Effective two-dimensional thickness for the Berezinskii-Kosterlitz-Thouless-like transition in a highly underdoped* $La_{2-x}Sr_xCuO_4$, Physical Review B **93**, 024519 (2016).

[7] Ying Xing, et al. *Quantum Griffiths singularity of superconductor-metal transition in Ga thin films,* Science **350**, 542 (2015).
